# Paclitaxel and cisplatin combined with concurrent involved-field irradiation in definitive chemoradiotherapy for locally advanced esophageal squamous cell carcinoma: a phase II clinical trial

**DOI:** 10.1186/s13014-022-02078-3

**Published:** 2022-06-09

**Authors:** Hui Li, Ying Fang, Dayong Gu, Mingyu Du, Zhi Zhang, Lei Sun, Guoren Zhou, Jinjun Ye

**Affiliations:** 1 Department of Radiation Oncology, Affiliated Cancer Hospital of Nanjing Medical University, Jiangsu Cancer Hospital/Jiangsu Institute of Cancer Research, No. 42, Baiziting, Nanjing, 210009 China; 2 Department of Oncology, Affiliated Cancer Hospital of Nanjing Medical University, Jiangsu Cancer Hospital/Jiangsu Institute of Cancer Research, No. 42, Baiziting, Nanjing, 210009 China; 3 Department of Thoracic Surgery, Affiliated Cancer Hospital of Nanjing Medical University, Jiangsu Cancer Hospital/Jiangsu Institute of Cancer Research, No. 42, Baiziting, Nanjing, 210009 China; 4 Department of Radiology, Affiliated Cancer Hospital of Nanjing Medical University, Jiangsu Cancer Hospital/Jiangsu Institute of Cancer Research, No. 42, Baiziting, Nanjing, 210009 China

**Keywords:** Paclitaxel and cisplatin, Involved-field irradiation, IMRT, Definitive chemoradiotherapy, Esophageal squamous cell carcinoma

## Abstract

**Purpose:**

This trial aims to explore the efficacy and safety of involved-field irradiation (IFI) combined with paclitaxel plus cisplatin as concurrent chemoradiotherapy for locally advanced esophageal squamous cell carcinoma (ESCC), under the premise of intensity-modulated radiotherapy (IMRT).

**Methods:**

Enrolled patients with locally advanced ESCC were treated with definitive concurrent chemoradiotherapy. IFI was administered adopting IMRT and the total dose was 61.2 Gy delivered in 34 fractions. On the first day of radiotherapy, the patients were treated with paclitaxel and cisplatin one cycle per month for 2 cycles followed by the same regimen in consolidation chemotherapy for two cycles. The primary endpoint of the study was the 2-year locoregional recurrence-free survival (LRFS) rate, and secondary endpoints included overall survival (OS), progression-free survival (PFS), and safety.

**Results:**

Between January 2018 and September 2020, 108 patients participated in the trial. 78.7% (85/108) of patients completed all 4 cycles of chemotherapy. The median follow-up of the surviving patients was 33.9 months (interquartile range, 29.2–41.1). The 2-year LRFS rate, as the primary endpoint, was 64.2%. In addition, the median PFS was 39.2 months, and 1-year and 3-year OS rates of 88.0% and 63.3%, respectively. Among the patients, out-of-field regional failure was seen in only 7 (6.5%) patients. Neutropenia grade 3 and 4 occurred in 21.3% and 37.0% of the patients, respectively.

**Conclusions:**

IFI using IMRT combined with paclitaxel and cisplatin concurrent chemotherapy for locally advanced ESCC yields encouraging local control and overall survival, but high hematological toxicity.

*Trial registration Clinical Trials* ChiCTR1800017039.

**Supplementary Information:**

The online version contains supplementary material available at 10.1186/s13014-022-02078-3.

## Introduction

Worldwide, there were an estimated 604,000 new cases and 544,000 deaths of esophageal cancer in 2020 [1], and there were about 320,000 new cases of esophageal cancer in China, making it the fourth leading cause of cancer death in China [2]. Ninety percent of these cases in China were squamous cell carcinoma [3]. The Radiation Therapy Oncology Group (RTOG) 8501 study established the critical role of concurrent chemoradiotherapy in inoperable locally advanced esophageal cancer. However, 41% of the patients in this study failed to complete the scheduled chemoradiotherapy as planned, 62.5% of the patients had acute grade 3 or above adverse events, and the 5-year OS rate was only 27% [4]. Although on the basis of this study, definitive radiotherapy concurrent with cisplatin plus fluorouracil is a standard modality for locally advanced esophageal cancer, its clinical application was restricted by the serious chemoradiotherapy-related toxicity.

As we all know, the limitation of the traditional two-dimensional irradiation field makes the actual dose and volume of the target area less than the prescribed dose level, which discourages it from protecting the adjacent normal tissues well and increasing the therapeutic dose. Nowadays, IMRT technology has been widely used in clinical practice. It can not only increase the dose to the target site but also protect the normal tissue around the target site, which is expected to improve the therapeutic gain ratio of radiotherapy. In this context, IFI is favored by some radiotherapy physicians because it reduces the side effects of radiotherapy without sacrificing the overall survival of patients [5–7].

With the exploration of new chemotherapy regimens, paclitaxel has received extensive attention from clinicians due to its radiosensitization in preclinical studies. A small-sample-size trial, RTOG 0113, compared paclitaxel-based regimens with the cisplatin plus fluorouracil regimen from the RTOG 9405 trial in dCRT for patients with locally advanced esophageal cancer, and showed that the paclitaxel-based chemotherapy regimen had an increasing trend in 1-year OS rate (76% vs 69%, *P* = 0.104) [8, 9]. Subsequently, in multiple phase II clinical studies of paclitaxel combined with cisplatin for concurrent chemoradiotherapy in esophageal cancer, it showed good tolerance, and the efficacy was not inferior to that of cisplatin plus fluorouracil [10–12]. In a phase II clinical study reported by Tang et al., IMRT combined with a 3-week schedule of paclitaxel plus cisplatin concurrent chemotherapy were arranged for the treatment of 76 patients with advanced esophageal squamous cell carcinoma. The median OS of this trial was 28.5 months, and the incidence of grade 3 and 4 leukopenia was 30.3% and 31.6%, respectively [10]. Overall, the efficacy of paclitaxel plus cisplatin in dCRT for esophageal cancer is satisfactory, and the side effects are acceptable.

In consideration of the above factors, we initiated this single-center, single-arm, open-label Phase II clinical study to investigate the efficacy and safety of a 4-week schedule of paclitaxel plus cisplatin combined with concurrent IFI for inoperable locally advanced ESCC. The protocol of paclitaxel plus cisplatin referred to the RTOG 0113 trial, with appropriate modifications due to the high toxicities reported by that trial.

## Materials and methods

### Patient eligibility

In this phase II prospective clinical trial, we recruited patients who met the following key criteria: 18–75 years of age; newly diagnosed, histologically confirmed squamous cell esophageal carcinoma, stage IIA to IVA (American Joint Committee on Cancer, 6th edition); Eastern Cooperative Oncology Group (ECOG) performance status 0–2; no serious hematopoietic, cardiac, pulmonary, hepatic or renal dysfunction; and adequate bone marrow function. Primary exclusion criteria included patients with complete esophageal obstruction, deep ulceration, or perforated hematemesis. Full inclusion and exclusion criteria refer to Additional file 1: File 1.

### Chemoradiotherapy

**Fig. 1:**
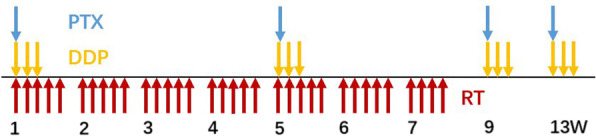
Study protocol diagram. PTX: paclitaxel; DDP: cisplatin; RT: radiotherapy

All patients had been treated with definitive chemotherapy with concurrent radiotherapy (Fig. 1). The total dose of radiotherapy using 6-MeV photons delivered by a linear accelerator was 61.2 Gy in 34 fractions. Patients were treated 5 days per week at 1.8 Gy/d. Target volumes were defined in accordance with the International Commission on Radiation Units and Measurements (ICRU) Report #62 [13]. The gross tumor volume (GTV) was contoured according to the chest positioning CT, esophageal barium meal imaging, and electronic gastroscopy, which included primary esophageal tumor (GTVe) and all metastatic lymph nodes (the short diameter of the lymph node local in the tracheoesophageal sulcus ≥ 5 mm, in the mediastinum ≥ 1 cm, and biopsy-confirmed metastatic lymph nodes). The clinical target volume (CTV) was defined as having a 3-cm cephalad and caudad margin beyond GTVe without radical margin. The planning target volume (PTV) provided proximal, distal, and radial margins of 1 cm around CTV. The field next to the spinal cord could be slightly adjusted to avoid excessive exposure. All patients were not given prophylactic irradiation.

The optimization scheme according to the dose-volume histogram is as follows: (1) tissue density inhomogeneity correction was used; (2) the 95% of the PTV received the prescribed dose; (3) the 95% isodose curve covered 99% of the PTV; (4) The maximum dose delivered to the PTV did not exceed the prescription dose by 10%; Dose limitations for the critical organs were as follows: (1) the mean lung dose was ≤ 13 Gy, and the V20 (percentage of the total lung volume receiving over 20 Gy) was ≤ 28%; (2) the mean heart dose was < 30 Gy; and (3) the maximum spinal cord dose was ≤ 45 Gy.

Radiation therapy was suspended when the patient developed the following conditions: WBC < 2.0 × 10^9^/L, ANC < 1.0 × 10^9^/L, PLT < 50 × 10^9^/L, or grade 4 nonhematological toxicity. A maximum 2-week delay to lower toxicity was permitted; otherwise, radiotherapy was terminated.

Concurrent chemotherapy was conducted on the first day of radiotherapy: paclitaxel (175 mg/m^2^), continuous intravenous drip for 3 h, day 1; cisplatin (25 mg/m^2^), i.v.drip., days 1 to 3. Cycles were duplicated every 4 weeks, for 4 courses altogether. If ≥ grade 3 hematological toxicity or ≥ grade 2 nonhematological toxicity (except for hair loss, nausea, and vomiting) were found and persisted, then chemotherapy was suspended until recovery. In principle, all patients were given the planned dose of chemotherapy, but the dose could be adjusted if necessary based on the most severe hematological or other toxicity. If a patient presented with multiple toxicities and the principles of dose adjustment differed from each other, the lowest dose was selected. The dose of cisplatin was decreased sequentially by 25%, and the dose of paclitaxel was decreased by 20% in sequence. Chemotherapy can be delayed for up to two weeks, with a maximum of dose reductions twice allowed. Complete dose adjustment principles refer to Additional file 1: File 2.

### Follow-up and data collection

Patients were followed-up for local recurrence and distant metastasis every 3 months during the first year, every 6 months for 2 additional years, and then once a year thereafter. Imaging tests including CT scan from the neck to the upper abdomen and esophagography were needed.

The primary endpoint was the 2-year LRFS rate in all recruited patients, in which LRFS was defined as the time from randomization to local/regional recurrence or death due to any cause. The secondary endpoints involved OS, PFS, and toxicity. OS was defined as the time from randomization until death. Toxicity was evaluated according to the National Cancer Institute Common Terminology Criteria for Adverse Events (AE) (NCI-CTCAE 4.0). In addition, PFS was defined as the time from randomization to recurrence, metastasis, or death (whichever occurs first).

The following clinical characteristics were obtained: of age, gender, tumor site, tumor length, stage, ECOG performance status, etc. Absolute lymphocyte counts (ALC) and absolute neutrophil counts (ANC) were recorded within the one week before dCRT. The nadir of ALC was the lowest, appearing within two months after the dCRT started. The neutrophil-to-lymphocyte ratio (NLR), was calculated by dividing the ANC by the ALC. The following radiotherapy-related variables were assessed based on the dose-volume histogram (DVH) parameters: mean heart dose (MHD), mean lung dose (MLD), V30 of heart, and V20 of lung.

### Statistical analysis

According to the previous studies [14, 15], the median PFS of radiotherapy combined with cisplatin plus 5-fluorouracil chemotherapy in esophageal cancer was 11.1 months. The necessary sample size to guarantee an improvement of 10 months in the median PFS, with a global alpha risk of 5%, power of 80%, an accrual period of 18 months, and 10% patient loss, was calculated.

Categorical variables were descriptively analyzed by frequency and proportion. Median and inter-quartile range (IQR) were used to summarize continuous variables. X-tile 3.6.1 software (Yale University, New Haven, CT, USA) was used to determine the best critical value of pretreatment NLR and ALC nadir. On this basis, the receiver operating characteristics (ROC) curve was used to determine the cut-off points for dosimetric parameters with ALC nadir as the state variable. Survival curves were plotted using the Kaplan–Meier method, with log-rank tests used to compare OS and PFS in subgroups, and hazard ratios (HR) were estimated using Cox regression models. SPSS 25.0 was used for data analyses.

## Results

### Baseline characteristics and treatment

A total of 108 eligible patients (excluding 2 patients who withdrew consent due to selecting surgical treatment) were recruited for this phase II trial in Jiangsu Cancer Hospital from January 2018 to September 2020, and their baseline characteristics are listed in Table 1. The majority were male (71%), and the median age of the enrolled patients was 65 years. The frequency of tumor clinical stage III (55%) is the highest and the median tumor length was 5 cm. The cervical or upper thoracic tumors were found in 57 (53%) patients.

**Table 1:** Baseline characteristics

Characteristic	N (%)
Age(y)	
Median (IQR)	65 (61–70)
Sex	
Male	77 (71%)
Female	31 (29%)
Stage (AJCC, 6th)	
IIb	19 (17%)
III	59 (55%)
IVa	30 (28%)
Tumor length, cm	
< 5	53 (49%)
≥ 5	55 (51%)
Tumor location	
Upper (< 25 cm)	57 (53%)
Middle (25-30 cm)	33 (30%)
Lower (> 30 m)	15 (14%)
Multiple primary	3 (3%)
ECOG performance score	
0	101 (94%)
1	7 (6%)
Chronic disease history	
Yes	39 (36%)
No	69 (64%)
Smoking history	
Former or current	58 (54%)
Never	50 (46%)
Drinking history	
Former or current	53 (49%)
Never	55 (51%)

*IQR* inter-quartile range

Among all patients, 2.8% (3/108) had interruptions during the radiotherapy period because of vomit, and 3.7% (4/108) have not finished radiotherapy. One patient’s radiation dose was 50.4 Gy because of the cerebral infarction. Two patients received the radiation dose of 54 Gy and 50.4 Gy respectively due to weak constitution. One patient’s radiation dose was 59.4 Gy because of his own decision. 85 (78.7%) patients completed all 4 cycles of chemotherapy, and the patients completing at least 3 cycles constituted 85.2%.

### Patterns of failure

As of April 1, 2022, 69 (63.9%) patients were alive (including 2 patients who were lost to follow-up at 24 months and 15 months after treatment, respectively) and 57 (52.8%) had no evidence of disease progression. Of the 51 patients who failed treatment (Table 2), 19 (17.6%) patients had only local/regional recurrence, 22 (20.4%) patients had only distant metastasis, and 3 (2.8%) had concurrent local/regional/distant failure. Another 7 (6.5%) patients failed due to other reasons, including cardiovascular and cerebrovascular accidents (1 case of acute myocardial infarction, 1 case of stroke), tumor complications (2 cases of upper gastrointestinal hemorrhage, 1 case of esophagotracheal fistula), second primary tumors (1 case of lung, 1 case of pancreas). The pattern of tumor local/regional recurrence was further analyzed. Among the 22 patients, 15 (13.9%) had recurrence within the irradiation field, and 7 (6.5%) had failure outside the irradiation field.

**Table 2:** Treatment failure pattern

First failure	N (%)
Distant metastasis only*	22 (20.4%)
Lung	10 (9.3%)
Liver	4 (3.7%)
Brain	4 (3.7%)
Bone	3 (2.8%)
Pleura	1 (0.9%)
Spleen	1 (0.9%)
Local/regional recurrence only	19 (17.6%)
Within the irradiation field	13 (12.0%)
Outside the irradiation field	6 (5.6%)
Concurrent local/regional/distant failure^&^	3 (2.8%)
Other reasons	7 (6.5%)

*Concurrent lung and liver metastasis were found in 1 patient, ^&^One of the patients had lymph node recurrence outside the irradiation field

### Survival

**Fig. 2:**
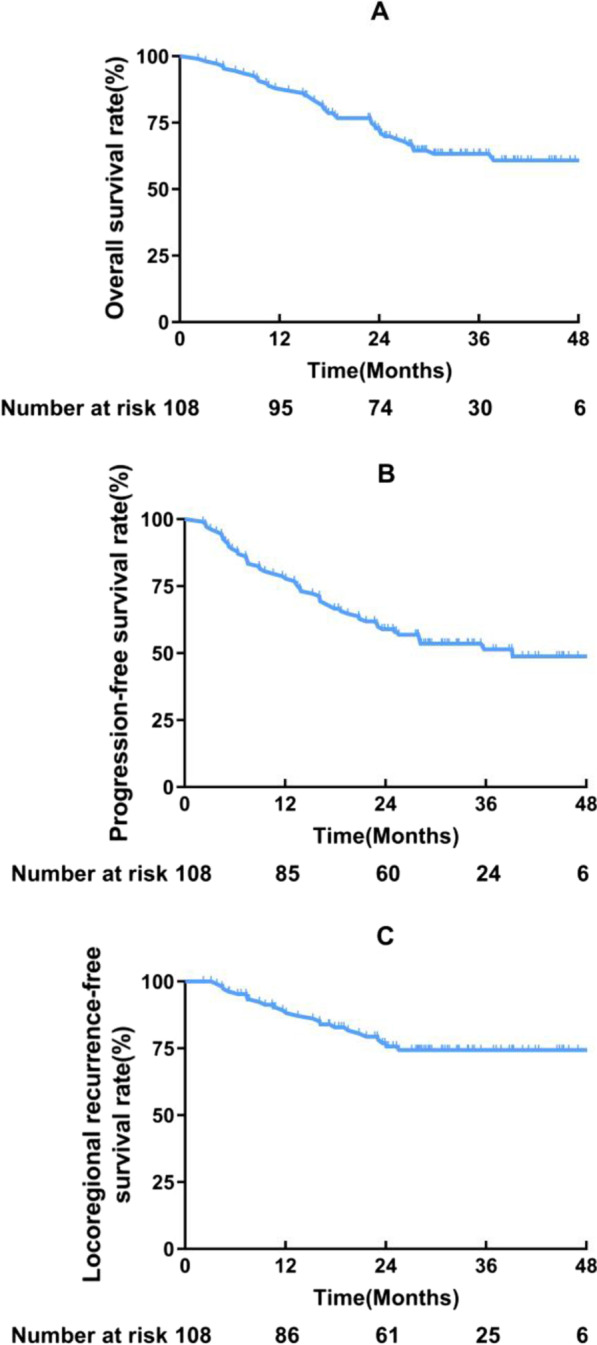
Survival curves of enrolled patients. **A** OS, **B** PFS, **C** LRFS

As of April 1, 2022, the median follow-up for surviving patients was 33.9 months (IQR, 29.2–41.1 months, 95% CI [30.7–37.0]) (Fig. 2). The 2-year LRFS rate for the primary endpoint was 67.3%. The median LRFS was 39.0 months, and the 1-year and 3-year LRFS rates were 81.2% and 53.6%, respectively. The median PFS was 39.2 months, with 1-year, 2-year, and 3-year PFS rates of 78.7%, 58.9%, and 51.4%, respectively. In addition, the 1-year, 2-year, and 3-year OS rates were 88.0%, 71.9%, and 63.3%, respectively (Fig. 2).

**Fig. 3:**
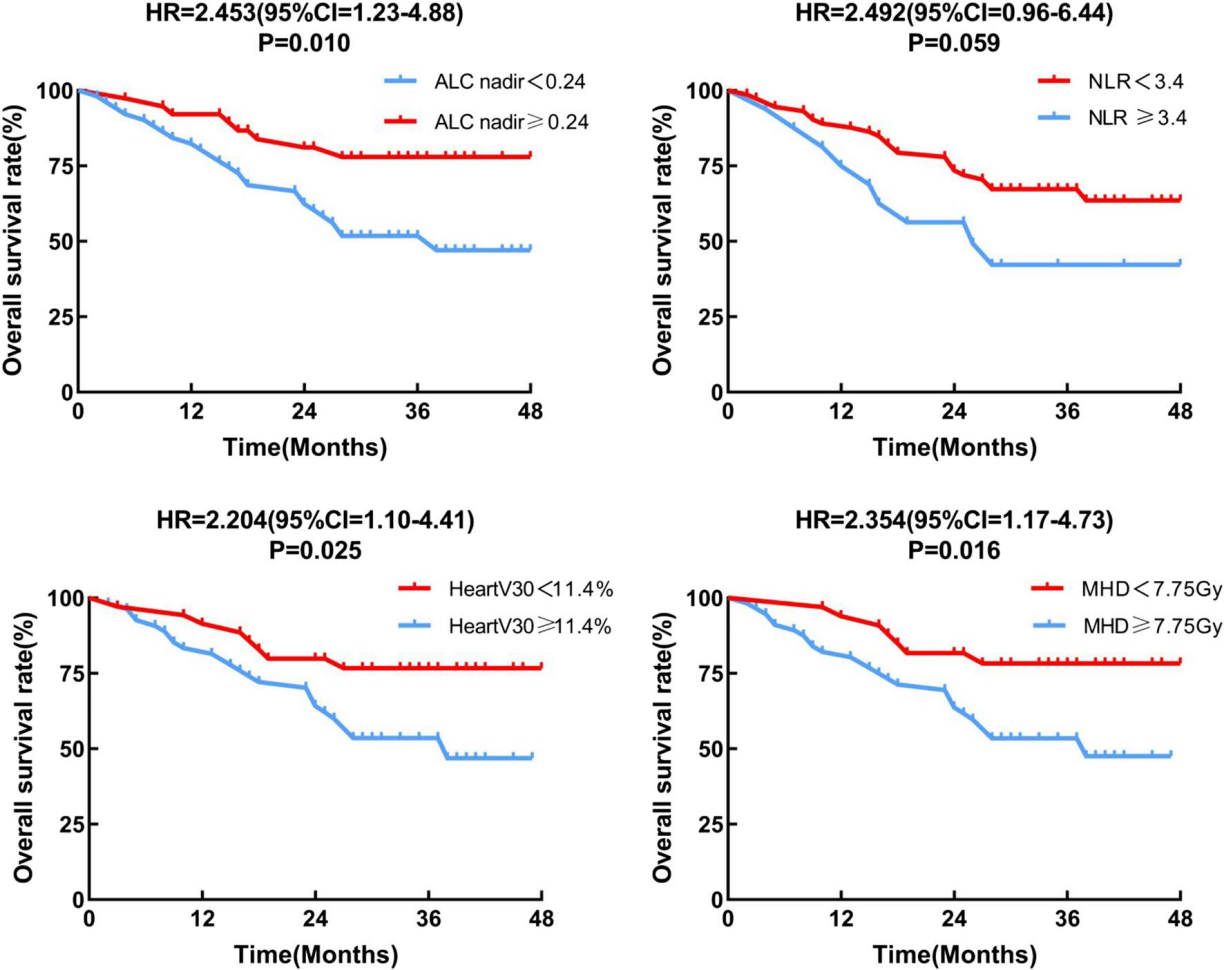
Overall survival curves for subgroups. Kaplan–Meier curves of overall survival for patients with ALC Nadir ≥ 0.24*109/L or < 0.24*109/L, for patients with Pretreatment NLR ≥ 3.4 or < 3.4, for patients with V30 of Heart ≥ 11.4% or < 11.4%, and for patients with MHD ≥ 7.75 Gy or < 7.75 Gy, respectively

**Fig. 4:**
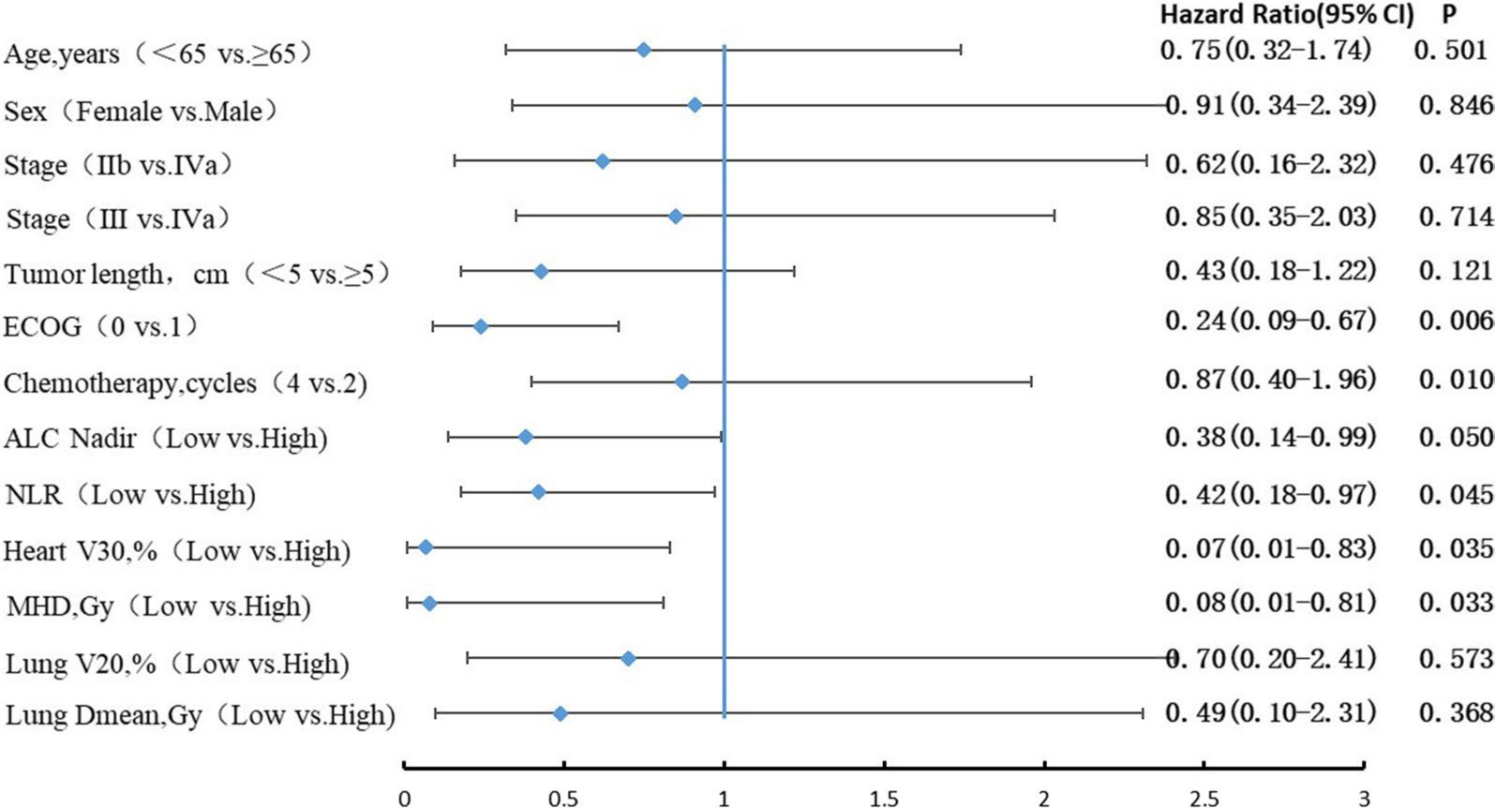
Subgroup analysis of overall survival

With reference to Fig. 3, the Kaplan–Meier curves for OS revealed that patients in the high ALC nadir group, low pretreatment NLR group, low V30 of heart group anandd low MHD group had longer OS. Based on the Cox regression multivariate analysis, the independent indicators of OS were ECOG performance, completion of chemotherapy, pretreatment NLR, ALC nadir, and dosimetric parameters of the heart (Fig. 4).

### Safety

All acute AEs were reported and listed in Table 3. The most frequent grade 3–4 acute AEs were neutropenia (63/108, 58.3%) and leukopenia (61/108, 56.5%), followed by thrombocytopenia (23/108, 21.3%) and anemia (11/108, 10.2%). Nonhematological AEs were mainly grade 1–2, among which acid reflux or vomiting (58/108, 53.7%), hair loss (58/108, 53.7%) had the highest incidence, followed by hiccups (30/108, 27.8%). Except for 1 case of grade 3 radiation pneumonitis, other radiation-induced injuries including esophagitis (84/108, 77.8%) and dermatitis (45/108, 41.7%) were all grade 1–2. There was no related toxicity for the liver, the kidney, and the heart recorded. In late toxicities, grade 1–2 in the esophagus and the lung/trachea were 1/108 and 5/108, respectively. No grade 3–4 AEs were tracked.

**Table 3:** Acute adverse events

AEs	N = 108 (%)		
	Grade 1–2	Grade 3	Grade 4
*Hematological*			
Leukopenia	32 (29.6%)	26 (24.1%)	35 (32.4%)
Neutropenia	25 (23.1%)	23 (21.3%)	40 (37.0%)
Anemia	65 (60.2%)	10 (9.3%)	1 (0.9%)
Thrombocytopenia	69 (63.9%)	18 (16.7%)	5 (4.6%)
*Nonhematological*			
Acid reflux/vomiting	58 (53.7%)	0	3 (2.8%)
Hiccup	30 (27.8%)	0	0
Fever	12 (11.1%)	0	0
Hair loss	58 (53.7%)	0	0
Muscular soreness	23 (21.3%)	0	0
Neurotoxicity	3 (2.8%)	0	0
*Radiation-induced*			
Dermatitis	45 (41.7%)	0	0
Esophagitis	84 (77.8%)	0	0
Pneumonitis	53 (49.1%)	1 (0.9%)	0

## Discussion and conclusion

The traditional concurrent chemoradiotherapy regimen has improved the local control rate and survival rate of inoperable locally advanced esophageal cancer to a certain extent, but the overall curative effect is still unsatisfactory, and further treatment strategies are urgently needed to be explored. Our trial mainly was designed to evaluate the efficacy and safety of IFI using IMRT combined with concurrent chemotherapy of paclitaxel and cisplatin in the treatment of locally advanced esophageal squamous cell carcinoma.

IMRT is considered to be superior to 3-dimensional conformal radiotherapy (3DCRT) in terms of the consistency of the prescribed dose and target volume, which provides powerful technical support for the stricter limitation of the dose of important organs such as the heart and lungs. A retrospective study showed that IMRT technology improved the 5-year OS rate of locally advanced esophageal cancer from 15 to 44% [16]. However, some retrospective studies suggested that the two radiotherapy options did not bring about differences in the efficacy of patients [17], whether related to the location of the tumor needs to be further confirmed. The use of IFI or elective nodal irradiation (ENI) in radical chemoradiotherapy for esophageal cancer has been controversial. In a prospective clinical study reported by Fudan Cancer Hospital in 2010 [5], under the 3DCRT technology, for patients with locally advanced esophageal cancer treated with concurrent chemoradiotherapy, the 3-year OS rate of patients receiving IFI was 41%, and the patients suffering regional recurrence outside the irradiation field accounted for only 8% of patients with treatment failure. Jiang et al. [18] reviewed several studies comparing IFI and ENI in chemoradiotherapy for esophageal cancer, and the results showed that IFI did not sacrifice the efficacy of patients, and the side effects of treatment are relatively reduced. In our trial, recurrence within the irradiation field occurred in 6.5% of patients, accounting for 13.7% (7/51) of patients who underwent treatment failure, and there was no case of long-term radiation-induced cardiac injury.

Also controversial is the optimal total radiation dose, which is a very hot issue and is very difficult to get a consensus among many investigators around the world. There was a trend to recommend high dose (> 60 Gy) in Asia and 50 Gy in Western countries. Many differences in patient and tumor factors could have produced those conflicting results. Recent multicenter, phase III randomized studies reported that 50 Gy is as effective as 60 Gy in ESCC, both in Europe (ARTDECO study) and China (Xu et al.) [19, 20]. In Xu et al.'s study, the incidence of radiation-related pneumonia of grade 3 or above in the 60 Gy group was higher than that in the 50 Gy group (7.5% vs 3.1%, *P* = 0.03). But there are still some points worth noting about these two studies. In the ARTDECO study, the difference in the radiotherapy target volume between the two experimental groups (the standard group included the primary tumor + regional lymph nodes, and the high-dose group only irradiated the primary tumor) may be related to the failure to obtain significant LRFS benefit in the high-dose group. In addition, in both studies, regional lymph nodes were selected for prophylactic irradiation, and the irradiation field was large, resulting in an increase in related radiotoxicity. In our study, based on the study design of involved field irradiation, we chose a total radiation dose of 61.2 Gy.

Additionally, with the development of proton radiotherapy, some studies had found that it can further reduce radiation lung and heart damage in patients with esophageal cancer [15]. These were still some small-sample studies and the efficacy and safety of proton radiotherapy for esophageal cancer need to be supported by data from further prospective studies, but it seems to provide a direction for our subsequent clinical research.

In routine clinical practice and worldwide trials, paclitaxel-based chemotherapy regimens have been widely adopted for concurrent chemoradiotherapy in patients with inoperable esophageal cancer. A prospective randomized controlled phase III clinical study (ESO-Shanghai1) conducted by Chen Yun et al. compared the efficacy of the concurrent chemotherapy regimen based on paclitaxel and the classical regimen of cisplatin plus fluorouracil in esophageal cancer patients receiving radical chemoradiotherapy [21, 22]. The results of the study showed that the option of paclitaxel and fluorouracil did not significantly improve the 3-year OS rate of patients compared with the regime of cisplatin and fluorouracil (55.4% vs. 51.8%), but the AE profiles significantly differed between the two regimens. The paclitaxel plus fluorouracil can significantly reduce the incidences of early gastrointestinal adverse reactions, anemia, and thrombocytopenia. Meanwhile, it had higher incidences of severe leukopenia, radiation dermatitis, and radiation pneumonitis. In another prospective phase II study of radical chemoradiotherapy for locally advanced esophageal cancer, compared with cisplatin plus fluorouracil regimen, paclitaxel combined with platinum group had lower incidences of grade 3–4 nausea, vomiting (5.88% vs. 35.29%, *P* = 0.003) and neutropenia (11.76% vs. 32.35%, *P* = 0.041) [12].

There is still a lack of high-ranking evidence on which paclitaxel-based chemotherapy regimen provides the best prognosis and the fewest adverse events. In this study, the median PFS was 39.2 months, which was significantly higher than previous similar studies involving paclitaxel and cisplatin regimen [10–12], and the 3-year OS rate was 63.3%, slightly better than the test group in the ESO-Shanghai1 study (55.4%). In terms of safety, the most common grade 3–4 acute adverse events were neutropenia (58.3%) and leukopenia (56.5%). The incidence was higher than that of some previous studies [11, 12], but similar to that reported by Tang et al. [10].and highly consistent with the results of a 3-group, multi-center, randomized phase III clinical trial (ESO-Shanghai2) led by Fudan University Affiliated Cancer Hospital [23].

High hematological toxicity not only requires the optimization of the combination regimen and the dose of chemotherapeutic drugs, but also poses a challenge to improve the performance of chemotherapeutic drugs themselves. As an improved new formulation, polymeric micellar paclitaxel (PM-paclitaxel) for injection had shown significant clinical benefits compared with the control group (ORR 50.33% vs. 26.4%, *P* < 0.0001) in the first-line treatment of non-small cell lung cancer, and its application prospect in esophageal cancer is worth looking forward to [24].

By subgroup analysis, we found that ECOG performance, the cycles of chemotherapy, pretreatment NLR, ALC nadir, and dosimetric parameters of the heart had significant effects on overall survival, suggesting that these clinical features might be prognostic factors. However, considering the impact of potential confounding factors, the results still need to be treated with caution. Fortunately, in our study, 78.7% of patients completed all 4 cycles of chemotherapy, and the vast majority of patients with low pretreatment NLR (89/108, 82%) or ECOG performance 0, which further verified the possibility of achieving higher LRFS and OS rates.

Several limitations should be considered when interpreting our findings. First, this study is a single-arm study, and no control group was set to exclude the interference of other factors. Second, the high hematological toxicity and frequent gastrointestinal reactions revealed by the study results should not be underestimated, and we did not assess the quality of life of the patients.

In conclusion, paclitaxel and cisplatin combined with concurrent involved-field irradiation in definitive chemoradiotherapy for locally advanced esophageal squamous cell carcinoma provides inspiring local control, overall survival, and high but manageable hematological toxicity.

## Supplementary Information

**Additional file 1.** File 1: Full inclusion and exclusion criteria. File 2: Complete dose adjustment principles.

## Data Availability

The raw data supporting the conclusions of this article will be made available by the authors, without undue reservation.

## Abbreviations

IFI: Involved-field irradiation
ESCC: Esophageal squamous cell carcinoma
IMRT: Intensity-modulated radiotherapy
dCRT: Definitive concurrent chemoradiotherapy
LRFS: Locoregional recurrence-free survival
OS: Overall survival
PFS: Progression-free survival
RTOG: Radiation Therapy Oncology Group
ECOG: Eastern Cooperative Oncology Group
GTV: Gross tumor volume
CTV: Clinical target volume
PTV: Planning target volume
NCI-CTCAE: National Cancer Institute Common Terminology Criteria for Adverse Events (AE)
HR: Hazard ratios
3DCRT: 3-Dimensional conformal radiotherapy
IQR: Inter-quartile range
ALC: Absolute lymphocyte counts
ANC: Absolute neutrophil counts
NLR: Neutrophil-to-lymphocyte ratio
DVH: Dosevolume histogram
MHD: Mean heart dose
ROC: Receiver operating characteristics
